# Supplementary material for: Effects of Probiotic NVP-1704 on Mental Health and Sleep in Healthy Adults: An 8-Week Randomized, Double-Blind, Placebo-Controlled Trial
Source: Nutrients. 2021 Jul 30;13(8):2660. doi: 10.3390/nu13082660 (PMC8398773; doi:10.3390/nu13082660)
Supplement: Supplementary file 1 [file nutrients-13-02660-s001.zip › Figure S1.pdf]

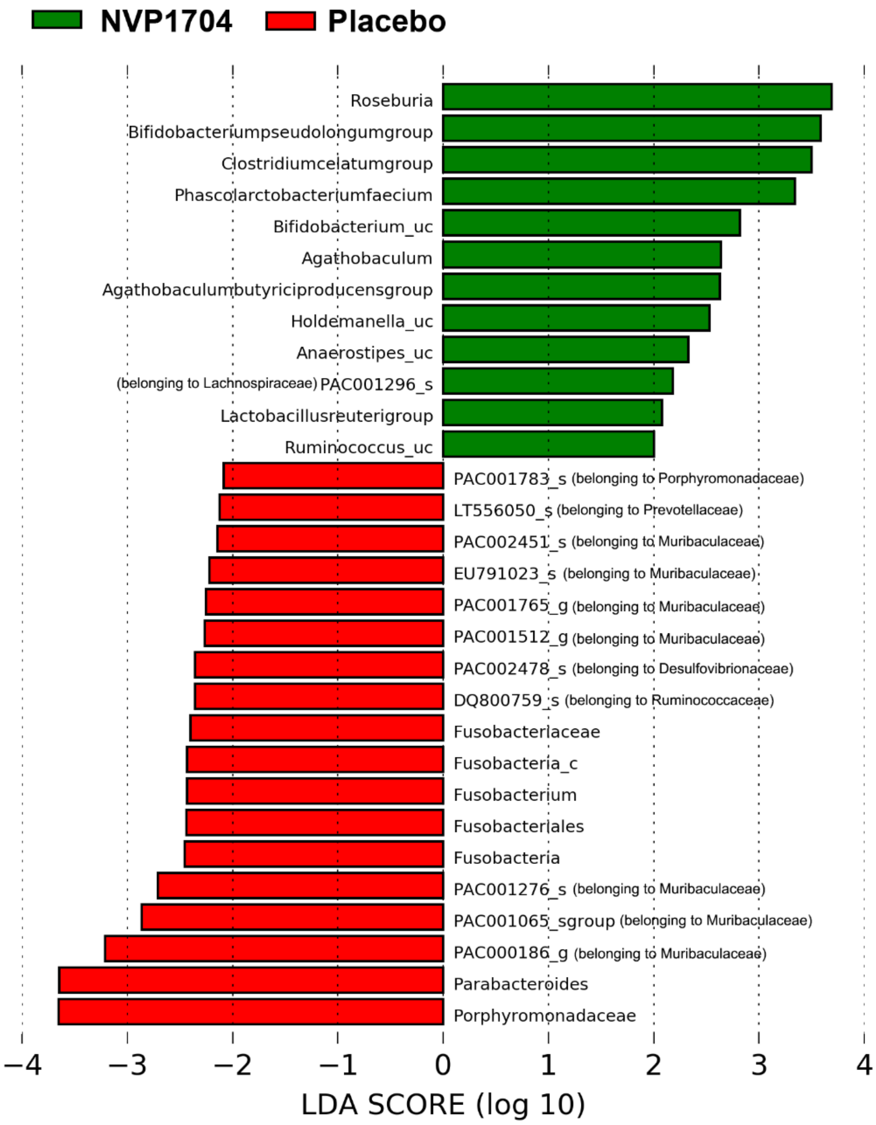

Figure S1. Linear discriminant analysis (LDA) score bar graph derived by LDA effect size analysis. LDA effect size (LEfSe) tool was derived strains significantly different between the two groups, assessed by the factorial Kruskal-Wellis test. The threshold logarithmic score set at 2.0 and ranked. Bacterial strains were described based on 16S rRNA sequencing data.
