# Supplementary material for: Effects of Probiotic NVP-1704 on Mental Health and Sleep in Healthy Adults: An 8-Week Randomized, Double-Blind, Placebo-Controlled Trial
Source: Nutrients. 2021 Jul 30;13(8):2660. doi: 10.3390/nu13082660 (PMC8398773; doi:10.3390/nu13082660)
Supplement: Supplementary file 1 [file nutrients-13-02660-s001.zip › Table S1.pdf]

Table S1. The gut microbiota composition ratio in the experimental and control groups after eight weeks of intervention.

Taxon name	Composition (%)	
	**Experimental group (n = 63)**	**Control group (n = 59)**
Phylum		
Actinobacteria *	2.52 (1.26-6.27)#	1.57 (0.52-4.68)
Bacteroidetes	26.89 (15.08-50.70)	31.53 (14.98-50.06)
Firmicutes	57.77 (40.35-70.86)	55.08 (29.26-70.68)
Proteobacteria	1.59 (0.74-3.87)	1.60 (0.45-5.44)
Tenericutes	0.02 (0.01-0.25)	0.01 (0.00-0.10)
Verrucomicrobia	0.02 (0.00-0.16)	0.02 (0.00-0.19)
Family		
Acidaminococcaceae	0.08 (0.00-0.68)	0.03 (0.00-0.55)
Akkermansiaceae	0.02 (0.00-0.16)	0.02 (0.00-0.15)
Bacteroidaceae	15.15 (5.96-37.22)	14.08 (5.63-34.09)
Bifidobacteriaceae *	1.84 (0.74-5.03)	1.16 (0.43-3.78)
Clostridiaceae	0.11 (0.03-0.40)	0.04 (0.02-0.25)
Enterobacteriaceae	0.25 (0.07-0.86)	0.35 (0.07-1.47)
Erysipelotrichaceae	0.60 (0.18-1.33)	0.50 (0.19-0.92)
Lachnospiraceae	12.88 (7.00-21.75)	12.24 (8.23-16.96)
Lactobacillaceae	0.15 (0.03-0.53)	0.08 (0.01-0.51)
Muribaculaceae	0.11 (0.05-0.30)	0.13 (0.05-0.48)
Peptostreptococcaceae	0.42 (0.10-2.80)	0.30 (0.10-1.38)
Porphyromonadaceae *	0.62 (0.19-1.95)	1.56 (0.33-3.58)
Prevotellaceae	1.10 (0.07-3.91)	1.05 (0.07-3.60)
Rikenellaceae	1.36 (0.21-3.27)	1.50 (0.35-4.74)
Ruminococcaceae	24.23 (13.32-35.43)	19.46 (9.19-37.00)
Veillonellaceae	0.66 (0.09-1.71)	0.43 (0.03-2.44)
Genus		
Akkermansia	0.02 (0.00-0.16)	0.02 (0.00-0.15)
Alistipes	1.36 (0.20-3.20)	1.38 (0.35-4.74)
Anaerostipes	0.41 (0.12-0.67)	0.39 (0.22-0.82)
Bacteroides	15.15 (5.92-37.07)	13.96 (5.56-34.00)
Bifidobacterium *	1.84 (0.74-5.01)	1.16 (0.43-3.76)
Blautia	1.55 (0.78-2.78)	1.26 (0.72-2.94)
Clostridium	0.11 (0.03-0.40)	0.04 (0.02-0.25)
Dialister	0.07 (0.02-0.95)	0.15 (0.01-1.27)
Escherichia	0.21 (0.07-0.64)	0.26 (0.06-1.17)
Eubacterium_g23	0.65 (0.09-3.17)	0.57 (0.08-2.60)
Faecalibacterium	7.17 (2.36-14.63)	6.11 (2.00-10.48)
Oscillibacter	1.38 (0.60-3.44)	1.68 (0.59-3.32)
PAC000661_g (Oscillospiraceae)	0.04 (0.01-0.53)	0.07 (0.01-0.84)
Parabacteroides *	0.61 (0.19-1.94)	1.56 (0.33-3.48)
Prevotella	0.32 (0.04-2.66)	0.62 (0.07-2.60)
Romboutsia	0.27 (0.07-1.62)	0.24 (0.05-0.95)
Roseburia *	2.25 (0.61-3.77)	1.30 (0.43-2.19)

Ruminococcus	0.27 (0.08-1.25)	0.20 (0.03-0.90)
Ruminococcus_g2	0.46 (0.05-1.43)	0.22 (0.04-1.99)
Subdoligranulum	1.59 (0.50-4.20)	1.88 (0.65-3.91)

Data are presented as median (percentile 25-75). One tailed Mann-Whitney test was performed. #$p < 0.05$ vs. placebo group.
